# A Method for the Immortalization of Newborn Mouse Skin Keratinocytes

**DOI:** 10.3389/fonc.2015.00177

**Published:** 2015-07-30

**Authors:** Brianna O. Hammiller, Taghrid Bahig El-Abaseri, Andrzej A. Dlugosz, Laura A. Hansen

**Affiliations:** ^1^Department of Biomedical Sciences, Creighton University, Omaha, NE, USA; ^2^Department of Dermatology, University of Michigan, Ann Arbor, MI, USA

**Keywords:** epidermal keratinocytes, immortalization, mouse skin, keratinocyte culture, orthopic skin grafting

## Abstract

Isolation and culture of mouse primary epidermal keratinocytes is a common technique that allows for easy genetic and environmental manipulation. However, due to their limited lifespan in culture, experiments utilizing primary keratinocytes require large numbers of animals, and are time consuming and expensive. To avoid these issues, we developed a method for the immortalization of primary mouse epidermal keratinocytes. Upon isolation of newborn epidermal keratinocytes according to established methods, the cells were cultured long-term in keratinocyte growth factor-containing medium. The cells senesced within a few weeks and eventually, small, slowly growing colonies emerged. After they regained confluency, the cells were passaged and slowly refilled the dish. With several rounds of subculture, the cells adapted to culture conditions, were easily subcultured, maintained normal morphology, and were apparently immortal. The immortalized cells retained the ability to differentiate with increased calcium concentrations, and were maintained to high passage numbers while maintaining a relatively stable karyotype. Analysis of multiple immortalized cell lines as well as primary keratinocyte cultures revealed increased numbers of chromosomes, especially in the primary keratinocytes, and chromosomal aberrations in most of the immortalized cultures and in the primary keratinocytes. Orthotopic grafting of immortalized keratinocytes together with fibroblasts onto nude mouse hosts produced skin while v-*ras^Ha^* infection of the immortalized keratinocytes prior to grafting produced squamous cell carcinoma. In summary, this method of cell line generation allows for decreased use of animals, reduces the expense and time involved in research, and provides a useful model for cutaneous keratinocyte experimentation.

## Introduction

Breakthroughs in cell culture methodology have facilitated molecular investigations of pathogenesis of diseases such as cancer. In the field of dermatological research, in particular, methods for culture of primary mouse keratinocytes have allowed for genetic and environmental manipulation of cultured keratinocytes for analysis of a variety of cancer-associated responses. Preparation and culture of primary newborn mouse keratinocytes, first published in 1975 [(1)] and then refined in subsequent publications using media with reduced calcium concentrations (2, 3), precipitated research in the fields of cutaneous keratinocyte biology and pathology. This method has over the last several decades contributed to the elucidation of signaling pathways, such as those involving calcium, protein kinase C, and the epidermal growth factor receptor in regulating normal epidermal functions and provided the molecular basis of our understanding of several skin diseases [reviewed in Ref. (4)]. In addition, primary keratinocytes have been critical to identification of oncogenic pathways that contribute to skin cancer development and progression [reviewed in Ref. (5)]. Lichti et al. (6) also described related methods for short-term culture of primary keratinocytes from adult mice and from follicular keratinocytes that expand the possibilities for keratinocyte research into additional fields.

Despite their contribution to our scientific understanding, current methods of mouse keratinocyte culture also present striking limitations. The short lifespan of primary mouse cultures, and their inability to be subcultured, generally requires that experiments be completed in a narrow several day window during the first week after plating (6). This generally necessitates euthanasia of large numbers of newborn mice for research purposes. For many laboratories, this translates into frequent labor-intensive preparation of keratinocytes for culture. In order to utilize cutaneous keratinocytes from the many transgenic and knockout mouse strains currently in use, genetically engineered mouse colonies must also be maintained, at great expense of time and money.

We present here a method for the generation of immortalized cell cultures from primary mouse keratinocytes that avoids some of the deficiencies of primary culture. Established colonies maintain many of the properties of the primary newborn mouse keratinocytes. For example, they are non-tumorigenic, differentiate with increased calcium, maintain a relatively stable karyotype no more aberrant than cultured primary keratinocytes, and produce epidermis upon orthotopic grafting onto nude mouse hosts. This method of cell line generation allows for decreased use of animals and reduces the expense and time involved in research.

## Materials and Methods

### Culture media

High calcium medium (HiCa) described in Ref. (6), which is necessary for primary keratinocyte adherence to the plates, was prepared from Lonza BioWhittaker Minimum Essential Medium Eagle (EMEM), with Earle’s balanced salt solution, non-essential amino acids, and l-glutamine without calcium chloride (Walkersville, MD, USA) supplemented with 8% fetal bovine serum (Atlas Biologicals, Fort Collins, CO, USA), 0.8% Pen Strep (Life Technologies, Grand Island, NY, USA), and 60 μM CaCl_2_. For regular maintenance of the cells, medium with approximately 0.05 mM calcium (LoCa medium) was prepared by supplementing EMEM with chelexed fetal bovine serum and 0.8% Pen Strep as described in Ref. (6). For some experiments, the medium was supplemented with epidermal growth factor or keratinocyte growth factor (KGF) (Life Technologies, Grand Island, NY, USA).

### Preparation and culture of primary newborn mouse keratinocytes

Preparation and culture of primary newborn mouse keratinocytes was performed as described in Ref. (6). In brief, the skin of euthanized newborn mice 0–3 days old was floated overnight on 0.25% Trypsin/EDTA (Life Technologies, Grand Island, NY, USA) at 4°C. The epidermis was removed using forceps. Chilled HiCa medium was added to the epidermis, and it was minced and triturated, then centrifuged. Cells were counted and plated at a density of approximately 2 × 10^5^ cells/cm^2^ in HiCa medium. Approximately 18 h later, the medium was removed, the dishes were washed with PBS, and the cells were refed with LoCa medium. For concurrent preparation of keratinocytes from mice with mixed genotypes, the abdominal skin was marked with an identifying number using a super-permanent marker prior to skinning, or skins were floated in individual dishes, with the tails reserved at skinning for genotyping. All experiments involving animals were performed in accordance with Creighton University’s Institutional Animal Care and Use Committee.

### Immortalization of primary cultures

Keratinocytes were prepared and plated as described above in HiCa medium in six-well plates (Corning Inc., Corning, NY, USA) at a density of one mouse/plate such that the cells were confluent within in a few days of plating. Approximately 18 h later, the medium was removed, the cells were rinsed with PBS, and the cells were refed LoCa medium containing 10 ng/ml KGF. The cells were refed with this medium every 2–3 days. The dishes were confluent in the first 2 weeks and then senesced, as indicated by a “pulled taffy” appearance. After several weeks of culture, viable cells were no longer apparent in the culture dishes. After approximately a month, occasional healthy keratinocytes were observed in the mostly empty dishes. These divided slowly to form small colonies of cobblestone-shaped keratinocytes morphologically similar to primary keratinocytes. When the colonies became confluent typically after close to 2 months in culture, the cultures were rinsed with PBS, incubated for several minutes with 0.25% Trypsin-EDTA (Life Technologies, Grand Island, NY, USA) until the cells detached from the dish, centrifuged for 3–5 min at 800 rpm (0.2 G), the supernatant removed, the cell pellet resuspended in HiCa medium with KGF, and the cells plated in fresh dishes without expanding the cultures (i.e., all the cells from one well were replated in a fresh well of the same size). Eighteen hours after replating, the cultures were washed with PBS and refed LoCa medium with 10 ng/ml KGF. The cultures were refed with this medium every 2–3 days until they were again confluent, which required several weeks. The cells were then trypsinized as described above, but this time the cultures were split 1:2. This process of subculture and regrowth to confluence was repeated until cells split 1:2 regained confluency within a few days. After several passages, the concentration of KGF in the medium was reduced to 5 ng/ml and then to 1 ng/ml after several more passages. Aliquots of cells were frozen following standard freezing protocols with 10% dimethylsulfoxide (DMSO) in LoCa medium beginning at P2–4.

### Immunofluorescence analysis

Immunofluorescence analysis of Keratin 14 (Covance Research Products Inc., Dedham, MA, USA), Keratin 1 (Covance Research Products Inc., Dedham, MA, USA), loricrin (Covance Research Products Inc., Dedham, MA, USA), Keratin 6 (Covance Research Products Inc., Dedham, MA, USA), or Ki67 (Abcam, Cambridge, MA, USA) was performed using standard techniques on cells fixed with 70% ethanol with an Alexa-fluor 488-conjugated secondary antibody (Life Technologies, Grand Island, NY, USA) and DAPI (4′,6-diamidino-2-phenylindole, Vector Laboratories, Burlingame, CA, USA) to identify the nuclei.

### Viral infection and immunoblotting

Immortalized cells were infected with a Cre recombinase-expressing adenovirus Cre recombinase-expressing (Sigma, St. Louis, MO, USA) or a control virus in polybrene and harvested 24 h later [as described in Ref. (7)] for immunoblotting with Erbb2 (Cell Signaling, Danvers, MA, USA), EGFR (Cell Signaling), GAPDH (Cell Signaling), or Actin (Sigma) antibodies.

### Grafting

Immortalized cells from FVB/N or CD-1 mice were infected with v-r*as^Ha^* retrovirus from Ψ-2 producer cell supernatant as described in Ref. (8). Viral infection was performed using diluted supernatant from Ψ-2 producer cells in the presence of 4 μg/ml polybrene. Four million v-r*as^Ha^*-transformed or uninfected immortalized keratinocytes were combined with 6 × 10^6^ primary fibroblasts and grafted onto the backs of athymic nude mouse hosts using a domed grafting chamber as described in Ref. (6, 9).

## Results

### Development of immortalized cells from primary mouse keratinocytes

**Figure 1:**
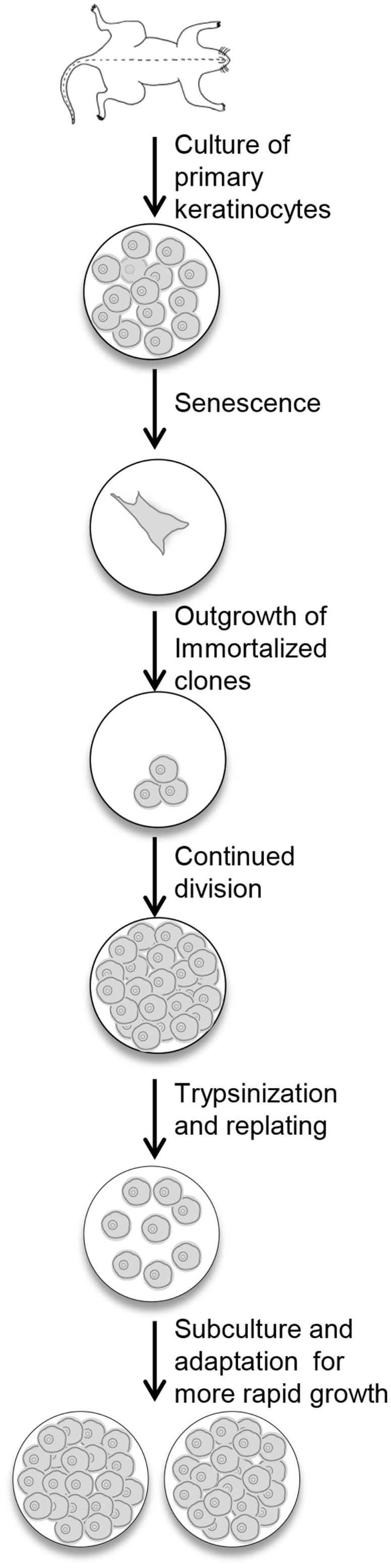
**Procedure for the immortalization of mouse keratinocytes**. Primary keratinocytes from newborn mouse skin are plated in medium containing KGF. The cells undergo senescence, and the culture dish will look empty. With continued refeeding, the dish will be repopulated with clonal outgrowths. Once these cells reach confluency, they are replated and slowly refilled the dish. With continued subculturing, the cells reach confluency faster, and can be split after trypsinization to expand the culture.

Newborn primary mouse keratinocytes were immortalized according to the protocol described in the Section “Materials and Methods” and illustrated in Figure 1. The process of immortalization and development of useful cell populations required several months and encompassed the steps of primary cell senescence, formation of single or small colonies of viable cells, repopulation of the dish by these viable cells, and finally acquisition of the ability to be subcultured and rapidly regrow to confluence (Figure 1). With each subculture, less time was required to regain confluency.

Immortalized cells were not successfully obtained in all cultures that were initiated. Contamination was a risk factor with these cells. Excellent sterile technique was essential for this protocol since it required the maintenance of the cells in the same dish for an extended period of time without contamination. The process required the addition of supplemental growth factors to the culture media. Either EGF or KGF supplementation (1–10 ng/ml culture media) allowed for the generation of immortalized cells, although KGF supplementation resulted in more frequent immortalization. Even with KGF supplementation, immortalized cells were not obtained from every well or dish plated, and a fraction of the cultures were lost because of contamination. In our experiments, six wells (one from each of six newborn mice) were plated for cell line generation from a mouse line. As shown in Table 1, the rate of survival of the cultures to at least P2 was 48% in a sample set of data from eight mice, with substantial variation among replicates (between one surviving well out of six plated and six wells out of six plated for individual mice).

**Table 1:** **Survival rates of cultures for cell immortalization**.

Mouse	Initial number of wells/mouse	Wells with surviving clones (no./mouse)*	Percent survival (%)
1	6	5	83
2	6	1	17
3	6	1	17
4	6	6	100
5	6	1	17
6	6	3	50
7	6	2	33
8	6	4	67

*Overall survival rate: 48% of wells*.

*To at least P2.

**Figure 2:**
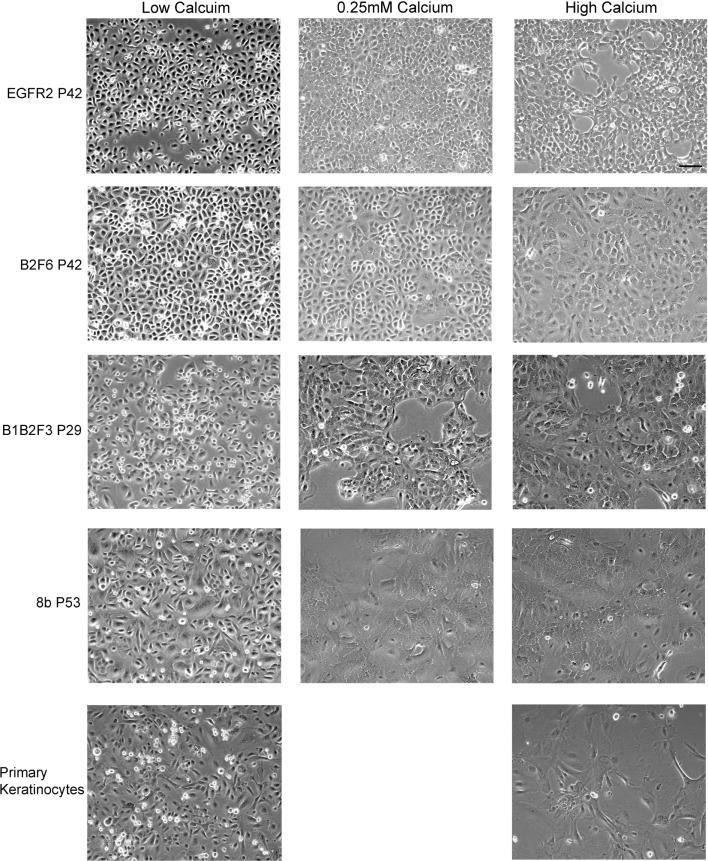
**Immortalized cells maintain morphologic characteristics of primary keratinocytes**. Immortalized EGFR2 cells generated from an *Egfr^fl/fl^* mouse on an FVB/N background, immortalized B2F6 cells generated from an *Erbb2^fl/fl^* mouse on an FVB/N background, immortalized B1B2F3 cells generated from an *Egfr^fl/fl^/Erbb2^fl/fl^* mouse on an FVB/N background, immortalized *Cdc25A^fl/fl^/Cre*^+^ (cell line 8b) keratinocytes on C57/Bl6 background, all at the indicated passage numbers (P), or primary keratinocytes from FVB/N mice were incubated in LoCa medium (left), or for 2 days in 0.25 mM calcium medium (middle), or HiCa medium (right). Phase contrast photos shown. Scale bar indicates 100 μm.

**Figure 3:**
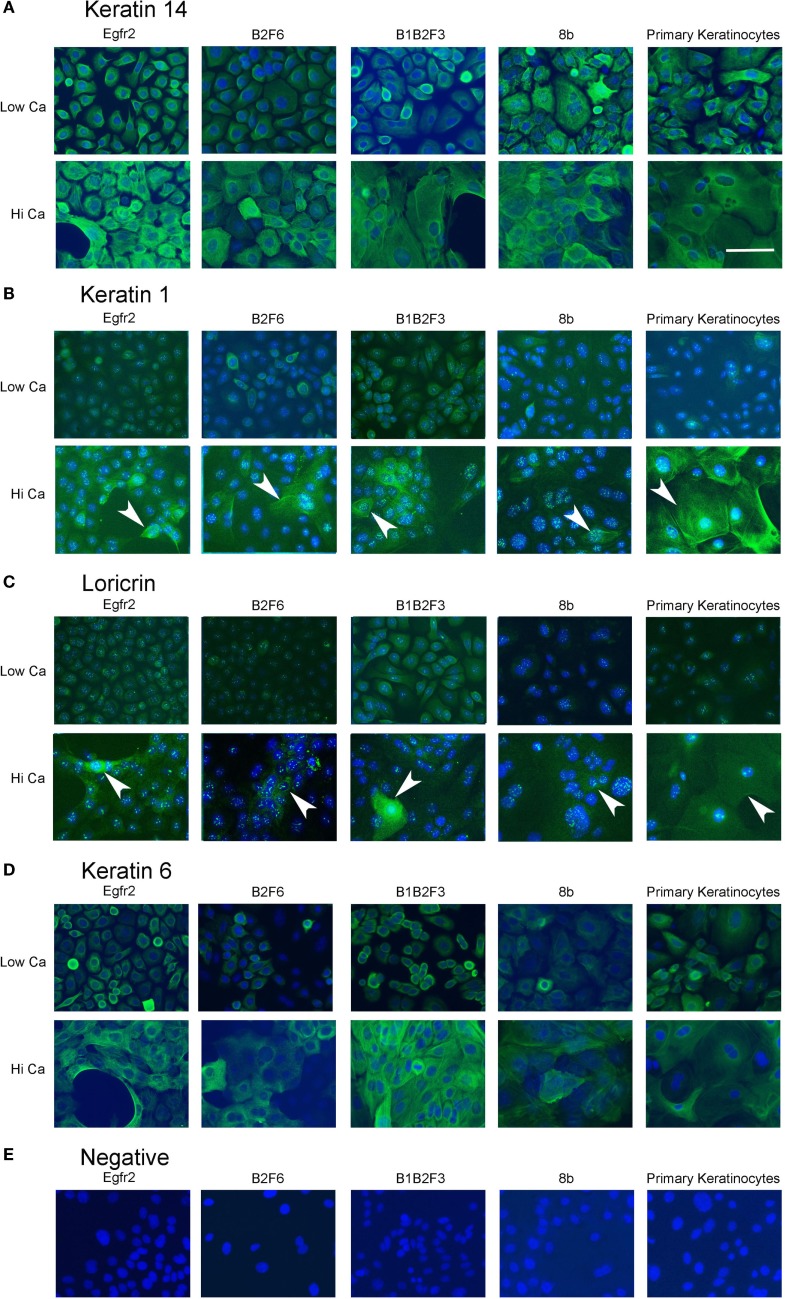
**Immortalized cells express keratinocyte markers and differentiate in response to increased calcium**. Immortalized EGFR2 cells (P 42), immortalized B2F6 cells (P42), immortalized B1B2F3 cells (P29), immortalized 8b cells (P53), or primary keratinocytes from FVB/N mice were treated with LoCa or HiCa media for 48 h, and immuno fluorescence was performed with the indicated antibodies (green) **(A–D)** or without primary antibody **(E)**. DAPI indicates nuclei (blue). Scale bar indicates 20 μm.

Cells that survived this process were apparently immortalized, given that they continued to grow well after as many as 100 subcultures. We have not attempted to passage the cells beyond this number. The cells were also able to survive freezing for later culture. These apparently immortalized keratinocytes had the regular cobblestone appearance typical of primary mouse keratinocytes even at high passage (Figure 2, left hand panels). Some of the cultures have been passaged over 100 times while maintaining typical keratinocyte morphology (not shown) and expression of the basal keratinocyte marker Keratin 14 (Figure 3A) and Keratin 6 (Figure 3D) compared to the negative control (Figure 3E). During early passages, cells were plated in HiCa media to promote adhesion. After multiple subcultures, the cells were successfully subcultured and replated without HiCa. KGF supplementation was gradually reduced to 1 ng/ml for maintenance, or removed for several days prior to their use in experiments.

**Figure 4:**
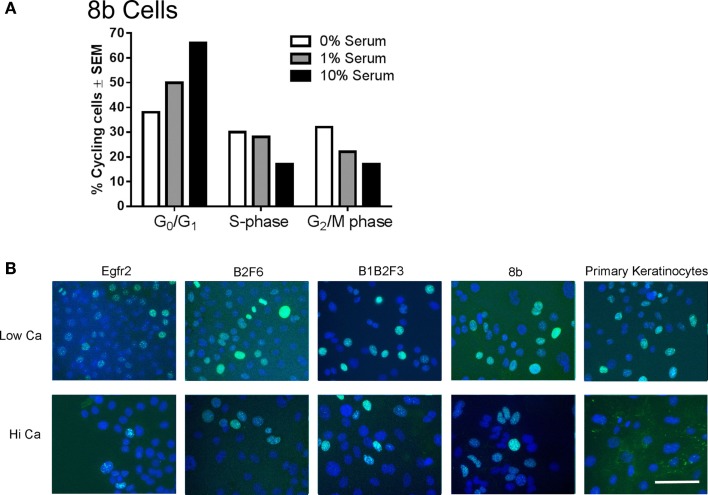
**Proliferation decreases in immortalized cells after serum deprivation or increased calcium**. **(A)** 8b cells were cultured for 48 h in media with the indicated serum concentrations and propidium iodide flow cytometry performed to assess cell cycle phase. *N* = 1. Experiment is representative of several that were performed. **(B)** Immortalized EGFR2 cells (P42), immortalized B2F6 cells (P42), immortalized B1B2F3 cells (P29), immortalized 8b cells (P53), or primary keratinocytes from FVB/N mice were treated with LoCa or HiCa media for 48 h, and immunofluorescence was performed for proliferation marker Ki67 (green). DAPI indicates the nuclei (blue). Scale bar indicates 20 μm.

Immortalized cells differentiated in response to increased calcium concentration. Forty-eight hours in increased calcium (HiCa medium or medium containing 0.25 mM calcium) resulted in morphologically evident squamous differentiation (Figure 2, middle and right-hand panels) in both primary keratinocytes (bottom row) and in cell lines, and induced the expression of the differentiation markers Keratin 1 and loricrin (Figures 3B,C, arrowheads). Some cell lines, for example 8b, were more sensitive to increased calcium (0.25 mM and HiCa) than were others (EGFR2, for example) (Figure 2). Cell proliferation, as detected by Ki67 immunofluorescence, ceased in primary keratinocytes after 48 h in HiCa medium (Figure 4B), while proliferation was substantially decreased in the cell lines in HiCa medium (Figure 4B). Twenty-four hours serum starvation of subconfluent cultures led to the accumulation of keratinocytes in G_0_/G_1_ phases, but not to complete cell cycle arrest, as shown by propidium iodide flow cytometry (Figure 4A).

**Figure 5:**
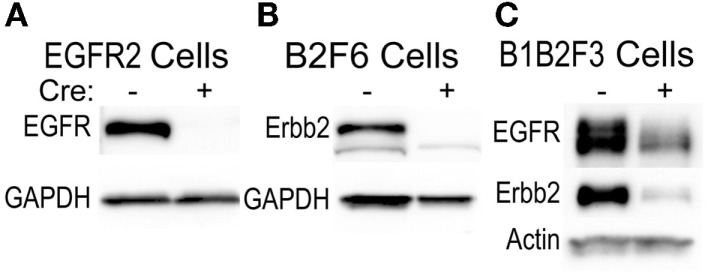
**Inducible gene deletion in immortalized cells**. The indicated cell lines from *Egfr^fl/fl^* FVB/N (EGFR2) **(A)**, *Erbb2^fl/fl^* FVB/N (B2F6) **(B)**, or *Egfr^fl/fl^*/*Erbb2^fl/fl^* FVB/N mice (B1B2F3) **(C)** were treated with a control virus or Cre recombinase-expressing adenovirus (Cre^+^), protein extracted 24 h later, and EGFR and Erbb2 levels assessed using immunoblotting.

This technique of immortalization of keratinocytes was successfully applied to mice of a variety of different backgrounds, including FVB/N (EGFR2, EGFR3, B2F6, B1B2F3 cells), C57Bl/6 (8B, F7 lines), and CD-1 (M3^−/−^, M4^−/−^, M3^+/+^, M4^+/+^) backgrounds. In addition, immortalized lines were generated from multiple transgenic mouse lines including v-*ras^Ha^* transgenic Tg.AC, *Egfr* null (M3^−/−^, M4^−/−^), *Egfr^fl/fl^* (EGFR2, EGFR3), *Erbb2^fl/fl^* (B2F6), *Egfr^fl/fl^/Erbb2^fl/fl^* (B1B2F3), and *Cdc25a^fl/fl^* (F7) mice. Generation of immortalized keratinocytes from mice with loxP insertion allowed for genetic deletion of the targeted locus upon adenoviral introduction of Cre recombinase in culture. Figure 5 shows decreased EGFR and Erbb2 receptor levels after Cre recombinase expression in lines generated from mice with floxed *Erbb2* (B2F6 cells in Figure 5B) (7), *Egfr* (EGFR2 cells in Figure 5A) (10), or both *Egfr* and *Erbb2* (B1B2F3 cells in Figure 5C) alleles.

### Karyotypic analysis of immortalized keratinocytes

To verify the identity of our immortalized mouse lines, karyotypes of several immortalized cell populations were obtained. All karyotypes were found to be of mouse cells, with some of the lines from male, indicated by the presence of Y-chromosomes, and some from female mice (Table 2). Karyotypes of primary mouse keratinocytes were also obtained from cells after several days of culture for comparison purposes. These primary keratinocytes had strikingly abnormal karyotypes, with dicentric and acentric fragments, multiple rearrangements, and increased and irregular numbers of chromosomes (Table 2). Some of these cells had more than 100 chromosomes with multiple rearrangements, and one spread was found to have more than 800 chromosomes. In contrast, immortalized cell populations were generally tetraploid or hyperdiploid (Table 2). B2F6 cells, immortalized cells generated from FVB/N mice with loxP sites flanking *Erbb2* exons (7), were also tested at passage 32 (P32) and P91, and found to have very similar karyotypes at early and late passage. At both passage numbers, a hyperdiploid karyotype was present in every spread. Thus, immortalized cells produced using this method are fairly stable both genetically and morphologically over many passages.

**Table 2:** **Karyotypes of immortalized and primary keratinocyte cultures**.

Cells	Passage number	Metaphase spread	Karyotype	Gender	Notes
Primary keratinocytes	P0	4 metaphase spreads with counts of >100 chromosomes, multiple rearrangements (MR)	Dicentrics and ascentric fragments	Y-chromosomes present	Multiple aberrations present
		1 spread, ~800 chromosomes w/MR	
		1 spread, ~75 chromosomes w/MR	
		1 spread, 40 XY chromosomes w/MR	
Cdc25A 8B	P11	4 metaphase spreads with counts of approx. 80	Tetraploid or hypertetraploid	Y-chromosomes present	Chromosomes appeared normal, high copy number
		3 metaphase spreads with counts of approx. 100	
Cdc25A F7	P39	6 metaphase spreads with counts of approx. 80	Tetraploid or hypertetraploid	No Y-chromosomes present	Chromosomes appeared normal, high copy number
		1 metaphase spreads with counts of approx. 100	
EGFR2	P49	2 metaphase spreads with counts of >100 chromosomes, multiple rearrangements (MR)	Multiple chromosome aberrations observed making karyotyping difficult		
		1 spread, ~90 chromosomes w/MR	
		1 spread, ~80 chromosomes w/MR	
		2 spreads, 50 chromosomes w/MR	
		1 spread, 40 chromosomes w/MR, metacentric seen	
B2F6	P32	3 metaphase spreads with counts of 63–68	Hyperdiploid	Y-chromosomes present	Many aberrant and normal chromosomes observed
		4 metaphase spreads with counts of 72–77	
B2F6	P91	5 metaphase spreads with counts of 65–69	Hyperdiploid	Y-chromosomes present	Many aberrant and normal chromosomes observed

### Orthotopic grafting onto nude mouse hosts

**Figure 6:**
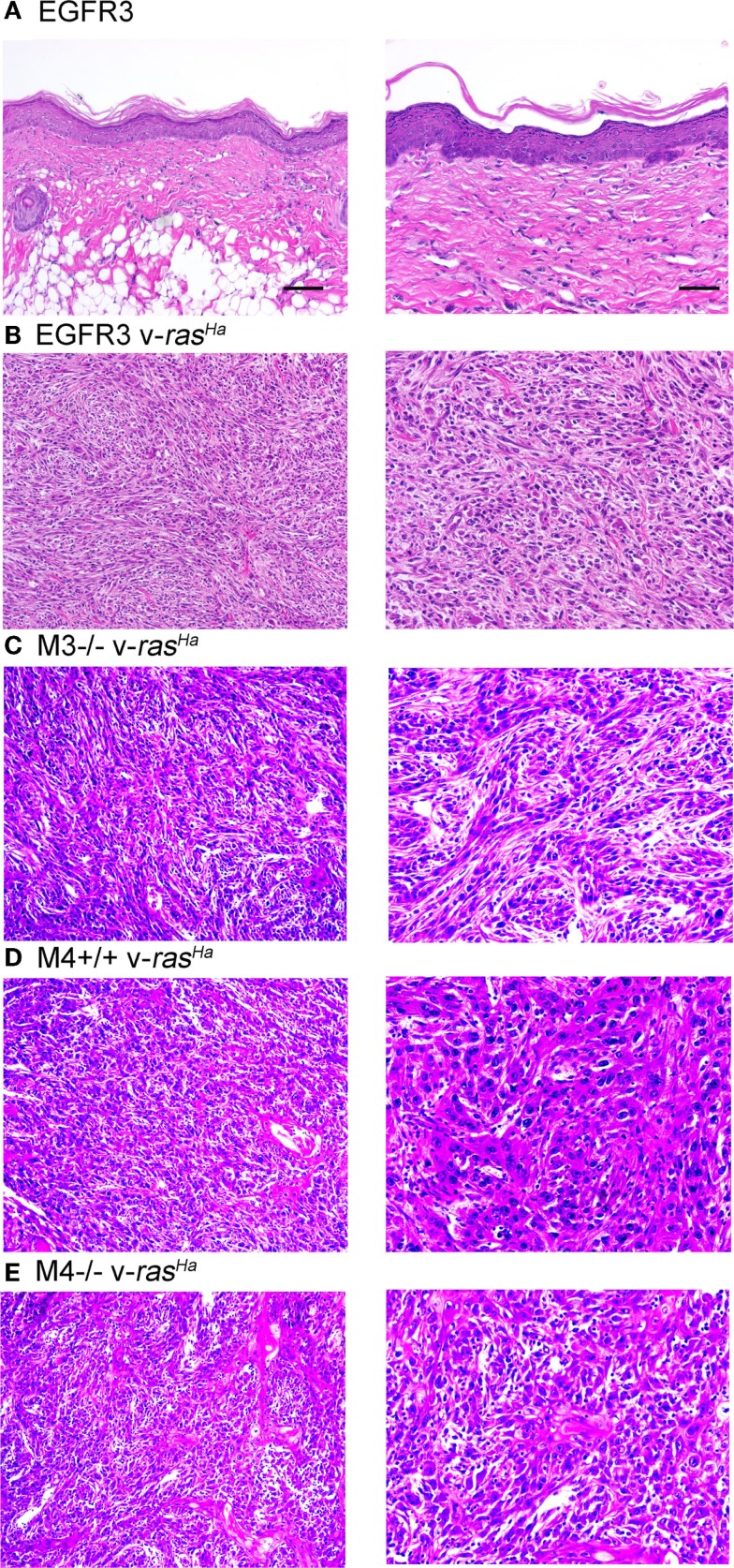
**Non-transformed immortalized keratinocytes produce epidermis in orthotopic skin grafts or squamous cell carcinoma following introduction of a v-*ras^Ha^* oncogene**. Immortalized EGFR3 line keratinocytes from an *Egfr^fl/fl^* mouse on an FVB/N background (*N* = 3) **(A)**, EGFR3 keratinocytes infected with a v-*ras^Ha^*-expressing retrovirus **(B)**, immortalized M3^−/−^ keratinocytes from an *Egfr^−/−^* CD-1 mouse infected with a v-*ras^Ha^*-expressing retrovirus (*N* = 6) **(C)**, immortalized M4^+/+^ keratinocytes from a CD-1 mouse infected with a v-*ras^Ha^*-expressing retrovirus (*N* = 6) **(D)**, or immortalized M4^−/−^ *Egfr*^−/−^ keratinocytes from an *Egfr*^−/−^ CD-1 mouse infected with a v-*ras^Ha^*-expressing retrovirus (*N* = 2) **(E)** were mixed with primary dermal fibroblasts and grafted onto the backs of athymic nude mice. Grafts were removed after 6 months, fixed, sectioned, and stained with hematoxylin and eosin. Images are representative of multiple grafts obtained for each group. Scale bars indicate 50 μm (left) or 100 μm (right).

Orthotopic grafting of immortalized keratinocytes with primary mouse fibroblasts onto nude mouse hosts was performed to determine their *in vivo* phenotype. As shown in Figure 6A, immortalized FVB/N (or CD-1, not shown) keratinocytes produced mildly hyperplastic epidermis when combined with normal fibroblasts, indicating that these immortalized cells were not transformed. v-*ras^Ha^* infection of the keratinocytes, which typically produces benign squamous papillomas on nude mouse hosts upon orthotopic grafting (11), resulted in squamous cell carcinoma development at the graft site (Figure 6B). Grafting of several other keratinocyte cell lines after v-*ras^Ha^* infection similarly produced squamous cell carcinoma (Figures 6C–E). Six of six grafts of either M3^−/−^ or M4^+/+^ cells after v-*ras^Ha^* transformation, the lines for which we have the largest *N*, produced squamous cell carcinoma (Figures 6C,D and not shown). The tumors were mostly poorly differentiated, with the *Egfr* null tumors exhibiting the most differentiation, in at least some microscopic fields, of all lines tested.

## Discussion

In our studies, we found that apparently immortalized mouse epidermal keratinocytes could be established from a variety of mouse lines using growth factor supplementation of the medium. These immortalized cells displayed many of the characteristics of primary keratinocytes, including a typical cobblestone morphology. The immortalized cells growth arrest and differentiate in response to increased calcium concentrations, although to a lesser extent than primary keratinocytes. Once the immortalized lines were established, they required much less time to maintain than repeated preparation of primary keratinocytes for experimentation. Immortalized lines could be reproducibly obtained from the long-term culture of the keratinocytes obtained from only a few newborn mice. The cells were relatively stable and grew for more than 100 subcultures. We have not attempted to culture them beyond this passage number. Thus, this procedure provides a virtually limitless supply of cells for experimentation and greatly reduces the number of animals needed for research.

Development of the lines required supplementation of the medium with a growth factor, either KGF or EGF, consistent with previous publications documenting clonal outgrowth of keratinocytes from primary culture upon EGF supplementation (12). We found that KGF was the more efficient growth factor for cell immortalization. The more vigorously dividing the primary cells, the more clones appeared, suggesting that the mitogenic effect of the growth factors may be an important stimulus allowing for cell immortalization in the cultures. Whether growth factors provided other stimuli necessary for immortalization is not known. The immortalized cells also survived when the growth factors were removed for several days prior to and during experimental manipulations. In order to decrease the selection pressures on the cells, our laboratory routinely prepares immortalized lines from floxed mice, and then introduces Cre recombinase to delete the targeted gene as part of the experimental protocol. As shown in Figure 5, this allows for generation of cells with substantially reduced levels of both EGFR and Erbb2 for experimentation, even though deletion of both receptors in the mouse does not allow for survival of the animals (unpublished observations).

The karyotypes of the immortalized cells were not normal. However, neither were the karyotypes of primary cultures of cells after only a few days in culture. After several days in culture, binucleate keratinocytes are common in primary mouse cultures (unpublished observations). The striking number of aberrations present in the primary keratinocyte cultures; which included a cell with 800 chromosomes, high variability among different cells, chromosomal fragments, and multiple rearrangements in all chromosomal spreads prepared; is still somewhat surprising. The immortalized cells also did not have normal karyotypes, mostly becoming tetraploid or hyperdiploid, and maintaining that karyotype for many passages, although they were more homogeneous and generally less aberrant than the primary cells. The immortalized population EGFR2 [from *Egfr^flfl^* mice, see Ref. (10)] had even more abnormalities, such that their karyotype was difficult to ascertain, while the CDC25A 8b and F7 populations [from *Cdc25a^fl/fl^/Cre*^+^ and *Cdc25a^fl/fl^/Cre^−^* mice, respectively, see Ref. (13)] were tetraploid but otherwise fairly normal. The cause of these differences is not known. As is clear from their karyotypes, this protocol produces a mixed culture rather than a true clonal cell line. However, the immortalized cells prepared in this manner have been successfully cloned via dilutional or ring cloning (not shown).

This protocol of mouse skin keratinocyte immortalization follows from a rich literature of primary keratinocyte culture experiments and from other examples of protocols able to immortalize primary mouse skin keratinocytes, most of which use feeder layers, growth factor or conditioned-media supplementation, and/or oncogenic or chemical carcinogen-induced transformation to produce immortalized lines (14–17). As mentioned previously, Yuspa et al. (12) previously reported EGF supplementation led to clonal outgrowth of primary keratinocytes, providing the initial impetus for examining the ability of growth factors to produce immortalized keratinocyte lines. Following from their work, Woodworth et al. utilized human papilloma virus (HPV) type 16 E6/E7 infection for immortalization, a protocol that produced transformed keratinocyte cell lines capable of generating malignant squamous cell carcinoma upon orthotopic grafting (18). The ability to generate tumors upon grafting depended on the genetic background of the immortalized cells, with FVB/N mice producing primarily squamous cell carcinoma (18). In contrast, spontaneous immortalization of FVB/N or other keratinocytes upon long-term KGF treatment in our hands produced non-transformed immortalized lines. Introduction of v-*ras^Ha^* followed by treatment with the carcinogen *N*-methyl-*N*’-nitro-*N*-nitroso-guanidine (MNNG) also allowed for the growth of colonies for at least a month in culture (18). Use of a feeder layer, as originally established by Rheinwald and Green for human keratinocyte culture (19), with the Morris protocol of adult mouse keratinocyte culture (20), also allows for spontaneous immortalization of mouse keratinocytes (21). Limitations of all protocols for skin keratinocyte immortalization, as for the protocol presented here, include (1) variation in the immortalized cells due to variation in the cells’ response, adaptation, and survival during the selection process and (2) diminished responses to growth arresting and differentiation signals. The nature of the cells selected for in various keratinocyte immortalization protocols is also not clear, although stem-like cells of the skin have a greater proliferative potential and are more likely to be clonogenic in culture (22).

In summary, the protocol presented here produced fairly uniform cultures of immortalized keratinocytes that retained many of the features of primary epidermal keratinocyte cultures. Other advantages of the present protocol include ease of use due to the elimination of the need for a feeder layer, the lack of carcinogen treatment or oncogene introduction such that the immortalized cells remain untransformed, and a relatively stable karyotype over many passages. We propose the use of immortalized keratinocytes obtained via this protocol as a means of reducing (1) the use of animals, (2) investigator time and labor, and (3) the expense in research into epidermal keratinocyte biology and pathology.

## Conflict of Interest Statement

The authors declare that the research was conducted in the absence of any commercial or financial relationships that could be construed as a potential conflict of interest.

## References

[1] Fusenig NE Worst PK Mouse epidermal cell cultures. II. Isolation, characterization and cultivation of epidermal cells from perinatal mouse skin. Exp Cell Res (1975) 93:443–57. 10.1016/0014-4827(75)90471-1 808420

[2] Hennings H Michael D Cheng C Steinert P Holbrook K Yuspa SH. Calcium regulation of growth and differentiation of mouse epidermal cells in culture. Cell (1980) 19:245–54. 10.1016/0092-8674(80)90406-7 6153576

[3] Yuspa SH Kilkenny AE Steinert PM Roop DR. Expression of murine epidermal differentiation markers is tightly regulated by restricted extracellular calcium concentrations in vitro. J Cell Biol (1989) 109:1207–17. 10.1083/jcb.109.3.1207 2475508 PMC2115750

[4] Mascia F Denning M Kopan R Yuspa SH. The black box illuminated: signals and signaling. J Invest Dermatol (2012) 132(3 Pt 2):811–9. 10.1038/jid.2011.406 22170487 PMC6340394

[5] Balmain A Yuspa SH Milestones in skin carcinogenesis: the biology of multistage carcinogenesis. J Invest Dermatol (2014) 134(e1):E2–7. 10.1038/skinbio.2014.2 25302469

[6] Lichti U Anders J Yuspa SH. Isolation and short-term culture of primary keratinocytes, hair follicle populations and dermal cells from newborn mice and keratinocytes from adult mice for in vitro analysis and for grafting to immunodeficient mice. Nat Protoc (2008) 3(5):799–810. 10.1038/nprot.2008.50 18451788 PMC6299324

[7] Madson JG Lynch DT Svoboda J Ophardt R Yanagida J Putta SK Erbb2 suppresses DNA damage-induced checkpoint activation and UV-induced mouse skin tumorigenesis. Am J Pathol (2009) 174(6):2357–66. 10.2353/ajpath.2009.080638 19406993 PMC2684199

[8] Roop DR Lowy DR Tambourin PE Strickland J Harper JR Balaschak M An activated Harvey *ras* oncogene produces benign tumours on mouse epidermal tissue. Nature (1986) 323:822–4. 10.1038/323822a0 2430189

[9] Yuspa SH Viguera C Nims R. Maintenance of human skin on nude mice for studies of chemical carcinogenesis. Cancer Lett (1979) 6:301–10. 10.1016/S0304-3835(79)80049-X 373879

[10] Maklad A Nicolai JR Bichsel KJ Evenson JE Lee TC Threadgill DW The EGFR is required for proper innervation to the skin. J Invest Dermatol (2009) 129(3):690–8. 10.1038/jid.2008.281 18830272 PMC2640443

[11] Dlugosz AA Hansen L Cheng C Alexander N Denning MF Threadgill DW Targeted disruption of the epidermal growth factor receptor impairs growth of squamous papillomas expressing the v-*ras*^Ha^ oncogene but does not block *in vitro* keratinocyte responses to oncogenic *ras*. Cancer Res (1997) 57:3180–8. 9242447

[12] Yuspa SH Koehler B Kulesz-Martin M Hennings H. Clonal growth of mouse epidermal cells in medium with reduced calcium concentration. J Invest Dermatol (1981) 76:144–6. 10.1111/1523-1747.ep12525490 7462678

[13] Yanagida J Hammiller B Al-Matouq J Behrens M Trempus CS Repertinger SK Accelerated elimination of ultraviolet-induced DNA damage through apoptosis in CDC25A-deficient skin. Carcinogenesis (2012) 33(9):1754–61. 10.1093/carcin/bgs168 22764135 PMC3514895

[14] Hager B Bickenbach JR Fleckman P. Long-term culture of murine epidermal keratinocytes. J Invest Dermatol (1999) 112(6):971–6. 10.1046/j.1523-1747.1999.00605.x 10383747

[15] Kaighn ME Camalier RF Bertolero F Saffiotti U. Spontaneous establishment and characterization of mouse keratinocyte cell lines in serum-free medium. In Vitro Cell Dev Biol (1988) 24:845–54. 10.1007/BF02623657 2457574

[16] Pera MF Gorman PA. In vitro analysis of multistage epidermal carcinogenesis: development of indefinite renewal capacity and reduced growth factor requirements in colony forming keratinocytes precedes malignant transformation. Carcinogenesis (1984) 5:671–82. 10.1093/carcin/5.5.671 6327109

[17] Weissman BE Aaronson SA. BALB and Kirsten murine sarcoma viruses alter growth and differentiation of EGF-dependent balb/c mouse epidermal keratinocyte lines. Cell (1983) 32:599–606. 10.1016/0092-8674(83)90479-8 6297803

[18] Woodworth CD Michael E Smith L Vijayachandra K Glick A Hennings H Strain-dependent differences in malignant conversion of mouse skin tumors is an inherent property of the epidermal keratinocyte. Carcinogenesis (2004) 25(9):1771–8. 10.1093/carcin/bgh170 15105299

[19] Rheinwald JG Green H Serial cultivation of strains of human epidermal keratinocytes: the formation of keratinising colonies from single cells. Cell (1975) 6:331–44. 10.1016/S0092-8674(75)80001-8 1052771

[20] Morris RJ Procedure for harvesting epidermal cells from the dorsal epidermis of adult mice for primary cell culture in “high calcium” defined medium. In: Leigh IM Watt FM, editors. Keratinocyte Methods. Cambridge: Cambridge University Press (1994). p. 25–31.

[21] Romero MR Carroll JM Watt FM. Analysis of cultured keratinocytes from a transgenic mouse model of psoriasis: effects of suprabasal integrin expression on keratinocyte adhesion, proliferation and terminal differentiation. Exp Dermatol (1999) 8(1):53–67. 10.1111/j.1600-0625.1999.tb00348.x 10206722

[22] Morris RJ Potten CS. Slowly cycling (label-retaining) epidermal cells behave like clonogenic stem cells in vitro. Cell Prolif (1994) 27(5):279–89. 10.1111/j.1365-2184.1994.tb01425.x 10465012

